# Synthesis, Crystal Structure, Theoretical Calculations, Antibacterial Activity, Electrochemical Behavior, and Molecular Docking of Ni(II) and Cu(II) Complexes with Pyridoxal-Semicarbazone

**DOI:** 10.3390/molecules27196322

**Published:** 2022-09-26

**Authors:** Violeta Jevtovic, Njood Alshammari, Salman Latif, Abdulmohsen Khalaf Dhahi Alsukaibi, Jamal Humaidi, Tahani Y. A. Alanazi, Fahad Abdulaziz, Samah I. Matalka, Nebojša Đ. Pantelić, Milica Marković, Aleksandra Rakić, Dušan Dimić

**Affiliations:** 1 Department of Chemistry, College of Science, University of Hail, Ha’il 81451, Saudi Arabia; 2 Department of Chemistry and Biochemistry, Faculty of Agriculture, University of Belgrade, Nemanjina 6, 11080 Belgrade, Serbia; 3 Faculty of Physical Chemistry, University of Belgrade, Studentski trg 12-16, 11000 Belgrade, Serbia

**Keywords:** Ni(II) complex, Cu(II) complex, NBO, methylene blue, DFT, biological activity

## Abstract

New Ni (II) and Cu (II) complexes with pyridoxal-semicarbazone were synthesized and their structures were solved by X-ray crystallography. This analysis showed the bis-ligand octahedral structure of [Ni(PLSC-H)_2_]·H_2_O and the dimer octahedral structure of [Cu(PLSC)(SO_4_)(H_2_O)]_2_·2H_2_O. Hirshfeld surface analysis was employed to determine the most important intermolecular interactions in the crystallographic structures. The structures of both complexes were further examined using density functional theory and natural bond orbital analysis. The photocatalytic decomposition of methylene blue in the presence of both compounds was investigated. Both compounds were active toward *E. coli* and *S. aureus*, with a minimum inhibition concentration similar to that of chloramphenicol. The obtained complexes led to the formation of free radical species, as was demonstrated in an experiment with dichlorofluorescein-diacetate. It is postulated that this is the mechanistic pathway of the antibacterial and photocatalytic activities. Cyclic voltammograms of the compounds showed the peaks of the reduction of metal ions. A molecular docking study showed that the Ni(II) complex exhibited promising activity towards Janus kinase (JAK), as a potential therapy for inflammatory diseases, cancers, and immunologic disorders.

## 1. Introduction

**Scheme 1:**
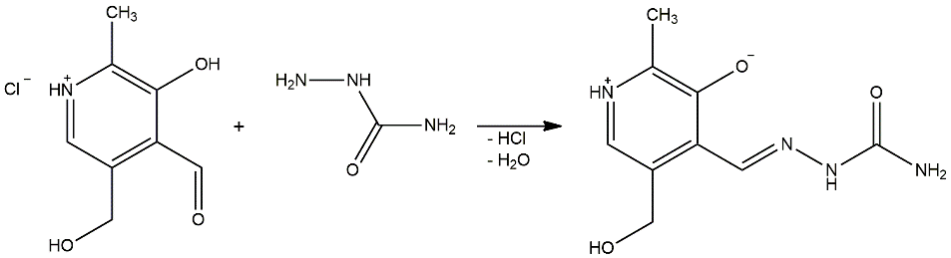
Synthesis of ligand pyridoxal-semicarbazone (PLSC).

**Figure 1:**
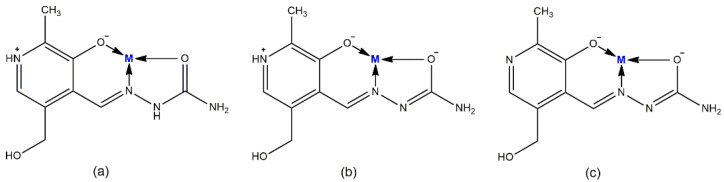
Mode of coordination of PLSC ligand in (**a**) neutral, (**b**) monoanionic-, and (**c**) dianionic- forms.

Pyridoxal-semicarbazone (PLSC) is a remarkable ligand due to the presence of various donor atoms [1]. PLSC is a combined ligand formed in the reaction of pyridoxal and semicarbazone, as presented in Scheme 1 [2]. The PLSC ligand has three donor atoms: oxygen from the phenolic hydroxyl group, hydrazine’s nitrogen, and oxygen from the amide group. In that way, the ONO ligand PLSC is formed. Based on the pyridoxal moiety (the form found in vitamin B6), this ligand has great potential in transition metal chemistry and the preparation of biologically active compounds [3]. The PLSC ligand can be coordinated in three ways, i.e., in neutral (H_2_L), monoanionic (HL^−^), and dianionic form (L^2−^) (Figure 1). In neutral form, the hydrogen atoms are present on both the pyridine and hydrazine nitrogen atoms. The aromatic OH group has the lowest pKa value and it is deprotonated in neutral form. Deprotonation of hydrazine hydrogen results in a monoanionic form of ligand PLSC, and full deprotonation occurs when both hydrogen atoms on the pyridine and hydrazone nitrogen atoms are removed. These various coordination modes of PLSC ligands allow the formation of a large number of newly obtained compounds. The form of the ligand depends on the pH of a solution. If the solution (the reaction mixture) is above 7, the base region accepts protons from the ligand PLSC and favors the ligand’s mono- or dianionic form. In the opposite situation, if the pH of the solution is less than 7, the ligand is predominantly in neutral form. Therefore, the pH of the solution dictates the coordination mode of the ligand.

In 2003, the first transition metal complexes with PLSC were produced, namely, [Pt (PLSC-H)Cl_3_] [1] and [CuBr_2_(PLSC)] [4]. Later, several papers on complexes, mostly copper and iron with the PLSC ligand, can be found [5,6]. Copper and cobalt complexes were predominantly synthesized, although there are also complexes with other transition metals. The following structures formed between copper and title ligand have been previously described in the literature: [Cu(PLSC)Cl_2_], [Cu(PLSC)(H_2_O) (SO_4_)]_2_·3H_2_O, [Cu_2_(PLSC)_2_(NCS)_2_](NCS)_2_, [Cu(PLSC)(NO_3_)_2_(CH_3_OH)], and [Cu(PLSC–2H]NH_3_·H_2_O, as published 2011 [3]. These copper complexes demonstrate excellent anticancer activity [7,8]. PLSC ligand complexes with cobalt are also numerous. Bis(pyridoxal-semicarbazone-k^6^, N^6^, O^2^, O^16^, N^21^, O^18^, O^25^) cobalt (II) nitrate dihydrate ([Co(PLSC)_2_](NO_3_)_2_, and the series Co(PLSC)(SO_4_)(H_2_O)_2_], [Co(PLSC-H)_2_]NCS·H_2_O, and [Co(PLSC-H)_2_]·H_2_O [9] are known in the literature. In the last decade, papers that describe the good catalytic properties of the transition metal complexes with PLSC have been published [10,11,12]. The deficiency of protons in the structures of different forms of coordinated ligand PLSC gives the possibility of using this ligand as a catalyst. It has been shown that organometallic copper and nickel complexes can be used as catalysts for C−S bond formation [13], and possible inhibitors of the main protease of SARS-CoV-2 [14].

This paper presents the results concerning the synthesis, crystal structure, and structural characterization of novel Ni(II) and Cu(II) complexes with pyridoxal-semicarbazone. The structures of both complexes were solved using X-ray crystallography and were additionally examined using IR spectroscopy. The intermolecular interactions governing the crystal structures of both compounds were analyzed by Hirshfeld surface analysis. The structure of Cu-pyridoxal-semicarbazone was optimized using density functional theory and further analyzed using the natural bond orbital approach. The antibacterial and photocatalytic activity of both compounds was determined and is discussed herein. Moreover, kinetic studies of the methylene blue decomposition in presence of these compounds were performed. The docking studies on Janus kinase were performed using molecular docking.

## 2. Results and Discussion

### 2.1. Crystal Structure

**Scheme 2:**
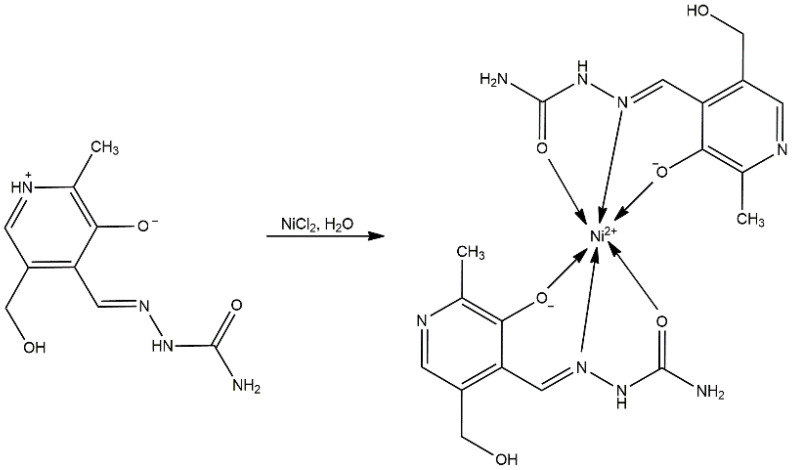
Synthesis of [Ni(PLSC-H)_2_]H_2_O.

The synthesis of the nickel complex was performed in an aqueous solution of NiCl_2_ and ligand at a molar ratio of 1:1. The pH of this solution was measured to be 4, which eventually led to the deprotonation of the ligand to its monoanionic form. Unexpectedly, a bis-ligand complex was obtained to some degree, as shown by X-ray crystallography (Table 1). The proposed formula of the newly obtained complex is [Ni(PLSC-H)_2_]H_2_O (Compound **1**), and the reaction route is shown in Scheme 2.

**Figure 2:**
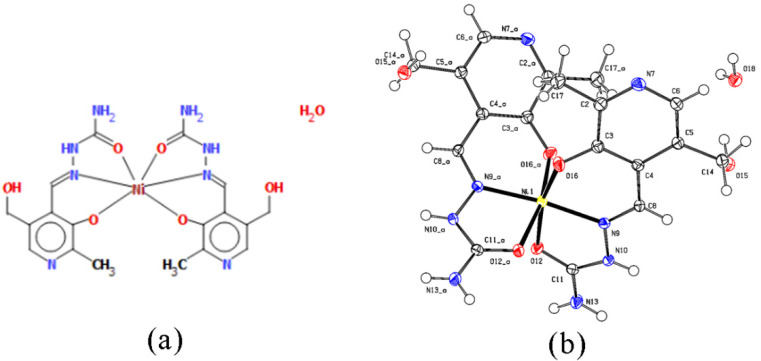
(**a**) Octahedral geometry of complex [Ni(PLSC-H)_2_]H_2_O and (**b**) the molecular structure of the complex [Ni(PLSC-H)_2_]H_2_O with the atom and ring-labeling scheme.

Nickel(II) complex is a bis-ligand complex with the monoanionic ligand PLSC in coordination with Ni^2+^. The geometry is octahedral (Figure 2), which is realized through two donor ONO systems. Confirmation of the monoanionic form of the ligand in the absence of hydrogen atoms on pyridine nitrogen is visible in the crystal structure. It is interesting to point out that this is the second known complex compound in which the deprotonation of the ligand occurs on pyridine hydrogen. The first complex in which this was observed is the chromium complex with PLSC [15]. However, it is more common to have a monoanionic form of the ligand obtained through the deprotonation of hydrazine nitrogen.

The bond lengths and angles of the most prominent bonds are presented in Table S1. Based on these results, it can be concluded that the bond lengths between nitrogen/oxygen atoms and Ni(II) are almost identical at around 2 Å. The structure is symmetric when two ligands are concerned, with identical bond lengths between donor atoms and metal. There is a slight distortion in angles, which leads to the deviation from the regular octahedron.

**Scheme 3:**
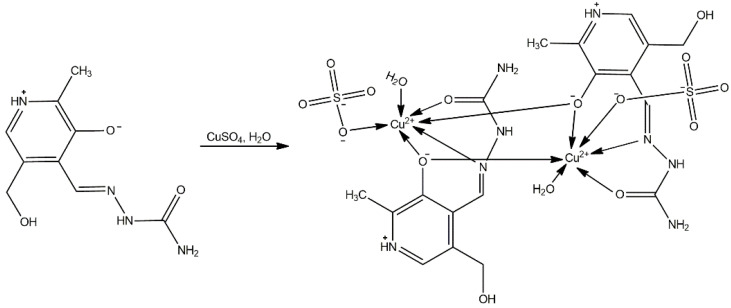
Synthesis of [Cu(PLSC)(SO_4_)(H_2_O)]_2_·2H_2_O.

The synthesis of the copper complex is presented in Scheme 3. The ratio of salt to ligand was 1:2 in favor of the ligand, and the bis-ligand complex was obtained accordingly. In addition, the pH of this reaction mixture was maintained as neutral, leading to ligand coordination in a neutral form (H_2_L).

**Figure 3:**
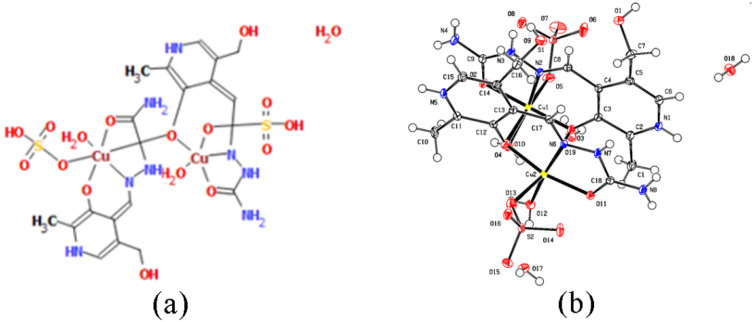
(**a**) Octahedral and square pyramid structure of two central Cu(II) in [Cu(PLSC)(SO_4_)(H_2_O)]_2_·2H_2_O complex and (**b**) the molecular structure of the complex [Cu(PLSC)(SO_4_)(H_2_O)]_2_·2H_2_O with the atom and ring-labeling scheme.

The copper complex is of a dimeric structure (Figure 3, Table 1), as was shown in the crystallographic analysis. The two unit cells are connected by a bridge between the copper of one unit cell and the oxygen of the phenolic hydroxyl group from the other unit cell. The proposed formula is [Cu(PLSC)(SO_4_)(H_2_O)]_2_·2H_2_O (Compound **2**). Each copper ion is in coordination with the tridentate (ONO) PLSC ligand in neutral form (H_2_L), and with one sulfate group and one water molecule. The coordination number is 6, and the geometry is octahedral for copper ions. The bond lengths between Cu and *O* atoms of the ligand are 1.90 (phenolic *O*) and 1.95 Å (carbonyl *O*). The bond length between Cu and *N* is 1.97 Å. The bonds between Cu and *O* with the additional species are 1.93 (water molecule) and 2.26 Å (sulfate group). The bond with phenolic *O* of the second ligand is 2.94 Å. The bond angles demonstrate a distorted octahedral geometry (Table S1).

### 2.2. IR Spectra of Compounds ***1*** and ***2***

**Figure 4:**
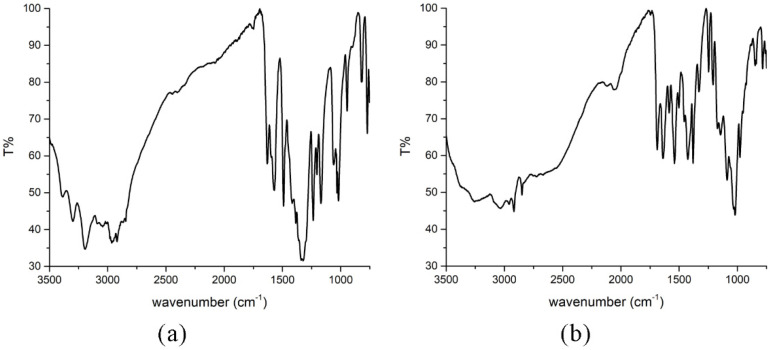
IR spectra of (**a**) [Ni(PLSC-H)_2_]H_2_O and (**b**) [Cu(PLSC)(SO_4_)(H_2_O)]_2_·2H_2_O.

The IR spectra of the obtained compounds are shown in Figure 4, while the spectrum of the ligand is presented in Figure S1. The presence/absence of specific bands in the spectrum allows the determination of the donor sites of the ligand. The band at 1675 cm^−1^ is found in the spectrum of the pure ligand and was assigned as the C=O stretching vibration [1], while in the spectra of complexes, this vibration is located at 1630 (Compound **1**) and 1639 (Compound **2**) cm^−1^. The shift is due to the elongation of this bond upon complexation. The C=N vibration is positioned at 1581 cm^−1^ in the spectrum of the ligand, as previously reported in [1], and it shifted upon complex formation to 1570 (Compound **1**) and 1540 (Compound **2**) cm^−1^. A similar thing was observed in the case of the Pt complex with the same ligand [1]. This shift is a consequence of donor bond formation between the nitrogen of the azomethine group and the metal ion [16,17,18]. The shift also depends on the chosen central metal ion [18]. The phenolic C−O vibration can be assigned to the peak at 1151 cm^−1^ in the spectrum of the ligand. This group is deprotonated in both compounds, which allows bond formation with a central metal ion. Therefore, these bands are shifted to 1019 (Compound **1**) and 1020 cm^−1^ (Compound **2**). The shift probably depends on the interaction strength. Protonation of pyridine nitrogen leads to a band between 2950 and 2800 cm^−1^ [3,19]. This band is observed in the spectrum of Compound **2** at 2849 cm^−1^. Based on this, it can be concluded that the ligand is in a zwitterion form in the case of Compound **2**, which is consistent with the crystallographic structure. This band is absent in the spectrum of Compound **1**. The broad bands above 3000 cm^−1^ can be assigned to the hydrogen bonds formed between ligands and water molecules [17,20,21,22]. The peak of the coordinated sulfate group is not visible in the spectrum due to the experimental conditions, although it is expected to be found around 620 cm^−1^. The peak that corresponds to the coordinated water molecule is found at 847 cm^−1^ in the spectrum of Compound **2**.

### 2.3. Hirshfeld Surface Analysis

**Figure 5:**
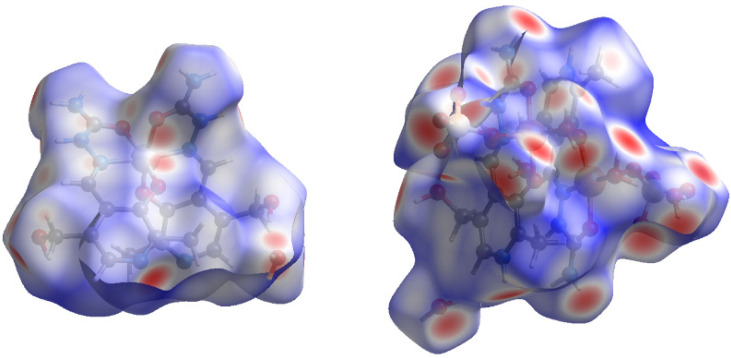
The Hirshfeld surface of compounds [Ni(PLSC-H)_2_∙H_2_O (**left**) and [Cu(PLSC)(SO_4_)(H_2_O)]_2_·2H_2_O (**right**).

The intermolecular interactions are important for stabilizing the crystal structure and Hirshfeld surface analysis was applied to investigate the experimentally obtained cif files. The fingerprint plots for the various contact atoms are presented in Figures S2 and S3, while the Hirshfeld surfaces are depicted in Figure 5. Both complexes have ligands surrounding the central metal ion, therefore interactions involving these ions between cells are not expected. These ligands are also characterized by multiple polar groups, such as phenolic *O*, amino, carbonyl, and pyridine nitrogen, which are responsible for forming the hydrogen bonds between ligands.

In the case of Compound **1**, two ligands surround the Ni(II) and electron donation occurs from the carbonyl group, azomethine nitrogen, and phenolic oxygen atoms. The rest of the polar groups are included in the formation of interactions with surrounding ligand molecules. Several red spots on the Hirshfeld surface (Figure 5, left) represent close contact with neighboring molecules. These contacts can be divided into two types of interactions. The first includes interactions in which Compound **1** is a hydrogen proton donor, such as those contacts involving amino and phenolic OH groups. On the other hand, electronegative atoms are positions in which Compound **1** acts as a hydrogen bond acceptor. The contribution to the total number of contacts allows the determination of the most numerous contacts, and these are shown in Figure S2. The highest contributions are for the H∙∙∙H and H∙∙∙O contacts, with the relative contribution of 44.8 and 23.3%. The hydrogen bonds are also formed with the nitrogen atoms, through H∙∙∙N (13.9%). The interactions with carbon atoms amount to 12.2%. These interactions can denote the H∙∙∙π contacts between a positively charged hydrogen atom and aromatic ring [23]. The possible interactions also include electronegative atoms (N and O) and carbon atoms, but the relative percentages are lower than 4%. Other interactions do not significantly influence stability.

The structure of Compound **2** is much more complex, as it includes two metal ions and two ligand molecules. Furthermore, two sulfate groups are part of the inner sphere of the compound, and therefore represent a source of oxygen atoms that can form interactions with neighboring ligands. The Hirshfeld surface of Compound **2** (Figure 5, right) contains much more red spots representing close contacts. As expected, the contribution of H∙∙∙O interactions is significantly higher than that of other contacts and it amounts to 56.5%. The multitude of groups containing hydrogen atoms is responsible for the weak H∙∙∙H interactions (34.9%). Surprisingly, the contribution of H∙∙∙N interactions is very low (2%), considering the structure of the ligand. When the crystallographic structure is analyzed, it can be seen that all of the nitrogen-containing groups point inward, which protects these groups from contact formation. The contributions from N∙∙∙C and C∙∙∙C are 1.4 and 1.1%. Based on this analysis, the stability of compounds is attributed to the hydrogen bond interactions between ligand molecules.

### 2.4. Structure Optimization and NBO Analysis

**Figure 6:**
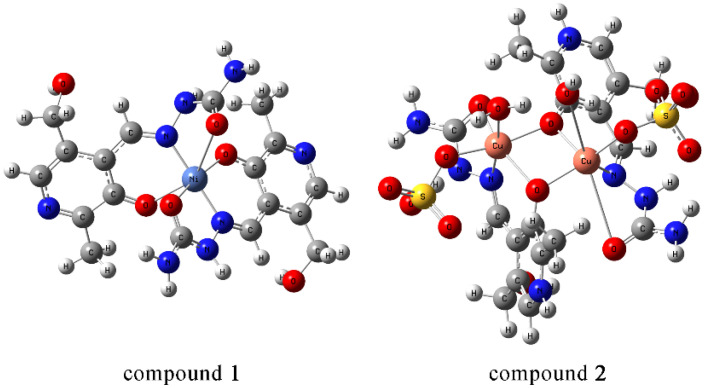
Optimized structures of Compounds **1** and **2** at the chosen level of theory (at B3LYP/6-31+G(d,p) for H, C, N, O, and S atoms and B3LYP/LanL2DZ for Ni/Cu).

The structures of Compounds **1** and **2** were optimized as described in the Materials and Methods Section of the manuscript. The optimized structures are presented in Figure 6. The same level of theory was previously applied to describing the structure of similar compounds [24]. The experimental and theoretical bond lengths and angles of the ligand were compared to calculate the mean absolute error (MAE), which represents the absolute difference between experimental and theoretical bond lengths and angles [25,26]. Tables S2 and S3 show the values of experimental and theoretical bond lengths and angles within the ligand. The MAE values for bond lengths and angles are 0.014 Å and 2.42°, respectively, proving that the selected theory level was appropriate for the investigated compound. These results are expected due to the extended delocalization within the structure of the ligand, as shown in the NBO analysis. Within the pyridine ring, the most prominent stabilization interactions include π(C−C) → π*(C−C), π(N−C) → π*(C−C), and π(C−C) → π*(N−C), with average energies between 50 and 60 kJ mol^−1^. The lone pair on the oxygen atom directly attached to the aromatic ring stabilizes the surrounding C−C bond for 37 and 57 kJ mol^−1^ (LP(O) → π*(C−C)). The electron density of the lone pair on the oxygen atom (part of the −CH_2_OH group) is also delocalized over the CH_2_ group and further within the aromatic ring (20 kJ mol^−1^). At the intersection between the aromatic ring and aliphatic chain, the stabilization interaction amounts to 8 kJ mol^−1^. Due to the presence of electronegative atoms with lone pairs and several double bonds, the aliphatic chain extends the delocalization of electrons. These stabilization interactions lead to shorter interatomic distances and increased stability of this part of the molecules. The strongest interaction includes the carbonyl oxygen atom and neighboring C−N bonds, LP(O) → π*(N−C) with energy between 92 and 104 kJ mol^−1^. Strong interaction can also be observed within the hydrazone group (LP(N) → π*(N−C)) with energy of 67 kJ mol^−1^. Within the amide group, the lone pair on the nitrogen is delocalized over the carbonyl group (LP(N) → π*(O−C)), at 200 kJ mol^−1^.

The optimization of Compound **1**’s structure led to the relaxation of the structure and slight changes in bond lengths and angles (Figure 6). In the experimental structure, the bonds between Cu and *N* were 1.9 Å, while in the optimized structure, they increased by 0.1 Å. The differences in experimental and theoretical bond lengths between Cu and carbonyl *O*/aromatic *O* were 0.1/0.3 Å. The angles deviated slightly from those presented in Table S1. The angle denoted as *O*12–Ni1–*O*16 was 167° in the crystalographic and 166° in the optimized structure. The rest of the angles increased by 5° on average. It should be kept in mind that these changes were expected because the optimization was performed for the isolated structure in a vacuum, while in crystal, these structures were stabilized by the intermolecular interactions, as previously explained in Section 2.3. It can be assumed that the co-crystalized water molecules stabilize the ligand structure through hydrogen bond formation with the methoxy group. The interactions between donor atoms and the Ni ion can also be quantified through the second order perturbation analysis. The interactions involving donor atoms varied significantly in strength. The weakest interaction energies were obtained for Ni and *N*. The interactions with aromatic *O* were 50 kJ mol^−1^ on average, while with carbonyl *O,* the stabilization of the system was much more pronounced (100 kJ mol^−1^).

During the optimization process, Structure **2** was also slightly distorted due to the complexity of the system (Figure 6). The bond lengths between Cu and oxygen atoms of the ligand in the theoretical structure were longer by 0.2 Å than in the experimental structure. The bond Cu-*N* bond lengths were 2.00 Å and 1.96 Å in the experimental and theoretical structures, respectively. The bond lengths between Cu and oxygen atoms in sulfate anion and water molecules were also well reproduced with an absolute difference of 0.2 Å. The change in some of the angles is notable, but this can be expected as discussed previously. The electron configuration of Cu ions is 4s^0.20^d^9.46^4p^0.28^ and 4s^0.22^3d^9.48^4p^0.36^, which is in keeping with the previous assumption that both Cu ions have a +2 charge. The interactions between oxygen atoms and Cu are much stronger than those between Cu and N. Stabilization energies for the Cu-*O* bond are between 13 (carbonyl *O*) and 100 kJ mol^−1^ (phenolic *O*). The stabilization energies with additional species are 40 (sulfate group) and 17 kJ mol^−1^ (water molecule). This theoretical analysis proved the applicability of the chosen level of theory for the theoretical description of Compounds **1** and **2**. These structures were used for further molecular docking studies.

### 2.5. Photocatalytic Activity

**Figure 7:**
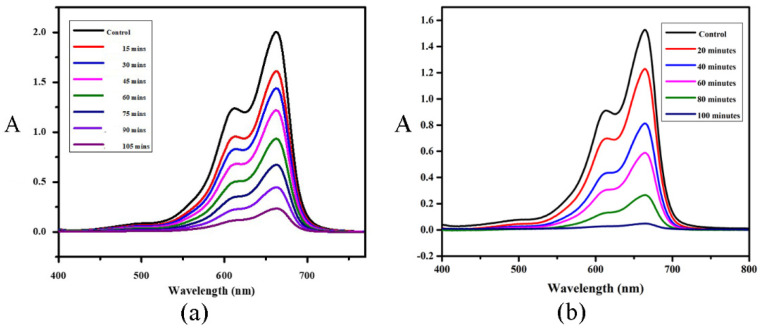
Photocatalytic reduction curves of MB after the addition of (**a**) [Ni(PLSC-H)_2_]∙H2O and (**b**) [Cu(PLSC)(SO_4_)(H_2_O)]_2_·2H_2_O under UV visible light irradiation.

Methylene blue (MB) is a dye used in different industries and is a main pollutant [27,28,29]. The harmful effect of this dye on living organisms, including humans, has been observed in several studies [30,31]. MB is a stable molecule due to its resonating structure and it represents a model compound for the photocatalytic degradation studies of various compounds. From the environmental point of view, it is very important to investigate the activity of compounds towards the degradation of these pollutants. The previous study on similar compounds proved that the degradation processes were based on the production of highly reactive free radical species [24]. The photocatalytic activity of Compounds **1** and **2** was investigated towards MB as explained in the Methodology Section. Figure 7 shows UV-VIS spectra of MB after the addition of the obtained compounds.

The UV-VIS spectrum of MB is characterized by two wide peaks at 664 and 614 nm, and these peaks were used to monitor the degradation process [32]. The UV-VIS spectrum of the ligand is presented in Figure S4. After the comparison, it can be concluded that the spectra of the ligand and MB do not overlap, which allows for the spectroscopic analysis of MB degradation. The shown spectra indicate the degradation process of MB in the presence of the obtained complexes. The degradation of MB after 105 min when Compound **1** was added was 90%, while, for the same period, the decrease in the absorbance of MB in the presence of Compound **2** was 98%. These compounds can be classified as very potent catalysts, which is expected based on previous research on similar ligand systems [24]. The absorbance at 664 nm was used for the preparation of the kinetic curves, as suggested in [33]. These results are presented in Figure S5 and they show that the reduction of MB follows a pseudo-first-order reaction kinetics for both complexes. The reaction rate in the case of Compound **1** was 2.2 × 10^−4^ s^−1^, while, for the second compound, the reaction rate was 3.6 × 10^−4^ s^−1^, which proves that the first compound is catalytically less active. The values of the reaction rates are similar to those published in previous research [24,33,34].

The possible mechanism of MB reduction may be multifactorial and complex. The significant photocatalytic performance can be attributed to the presence of nanosized particles with a large surface area that contains multiple active sites for the interaction and removal of MB. Other factors, such as crystallinity, size, and morphology, may also be important. As previously mentioned, the formation of reactive oxygen species due to the use of UV light and catalytic activity on the surface of compounds are probable mechanistic pathways. The degradation route of MB includes several possible mechanisms, leading to the removal of chlorine, nitro, and sulfate ions, the formation of benzothiazole and phenol, and ultimately water and carbon dioxide, as shown previously [35]. The assumption that radical species are formed in the presence of obtained compounds is explored in the following section with the formation of a fluorescent oxidized molecule of dichlorofluorescein.

### 2.6. Antibacterial Activity

The increase in bacterial infections over the past few years and the growing resistance of such bacteria to available drugs pose a great threat to human health and the environment. To encounter such a growing issue, several efforts have been made to develop new materials with significant antibacterial properties [36,37,38,39]. However, the biocompatibility of such materials plays a critical role in guaranteeing safety for practical applications. It must be noted that metal-based nanomedicines are of great importance; however, these can be endured up to certain limits by normal cells. Compounds **1** and **2** were tested against *E. coli* and *S. aureus* bacteria strains, as examples of Gram-negative and Gram-positive strains. The positive control included kanamycin, an aminoglycoside bactericidal antibiotic. The observed zones of inhibition are shown in Table 2.

Based on these results, it can be concluded that both compounds are active toward both bacterial strains in a concentration-dependent manner. In the case of Compound **1**, the zone of inhibition increased from 10 to 21 mm for *E. coli* and 12 to 24 mm for *S. aureus* when the concentration increased from 0.25 to 2 mg mL^−1^. The activity of Compound **2** is somewhat higher, although in a similar range. The zone of inhibition increased from 9 to 24 mm for *E. coli* and from 11 to 26 mm for *S. aureus* when the concentration of Compound **2** increased from 0.25 to 2 mg mL^−1^. The zone of inhibition from kanamycin was 15 mm for a concentration of the active compound of 0.25 mg mL^−1^. These results show that both compounds can be considered active towards selected bacterial strains, with Compound **2** being more active. These compounds’ activities were higher than those of Zn(II) and Cu(II) complexes with similar ligands, as previously published [24]. It was shown that thiosemicarbazone and semicarbazone ligand-containing complexes are active toward *S. aureus*, *E. coli*, and *S. epidermidis*, with the pronounced effect of metal ions, which is consistent with the findings of this study [40,41,42]. The enhanced antibacterial activity could be attributed to the difference in composition of both bacterial cell walls. *E. coli,* as a Gram-negative bacterium, has a thin peptidoglycan layer with an additional layered structure called an outer lipopolysaccharide membrane (LPS), whereas, *S. aureus,* as a Gram-positive bacterium, only has a thick peptidoglycan layer and no additional layers. A cell wall enables a bacterial cell to develop a definite shape/structure. Moreover, the cell wall plays a vital role for bacteria as it acts as a barrier against some particles and cells or it may act as a target for several antibiotics. Therefore, it can be suggested that the formation of free radicals is one of the mechanisms fors the antibacterial activity of these compounds, which is investigated in the following paragraphs.

### 2.7. Minimum Inhibition Concentration (MIC)

The effectiveness of the obtained compounds was also analyzed by determining the MIC values, which refer to the minimal quantity of the sample that prevents the growth of the relevant microorganisms. The concentration of Compounds **1** and **2** varied between 60 and 20 μg mL^−1^ and the inhibition of *E. coli* and *S. aureus* bacteria growth was followed (Table 3). The MIC values for Compound **1** were 30 and 40 μg mL^−1^ against *S. aureus* and *E. coli*, respectively. In the case of Compound **2**, these values were 20 and 30 mg mL^−1^, which is lower than for the previous compound. These results follow the previous experiments and can be attributed to the formation of reactive oxygen species. The MIC values of both compounds are comparable to the positive control, chloramphenicol, which classifies them as moderately to highly active compounds. The obtained results are also comparable to similar compounds found in the literature [15,24].

### 2.8. Production of Reactive Oxygen Species

**Figure 8:**
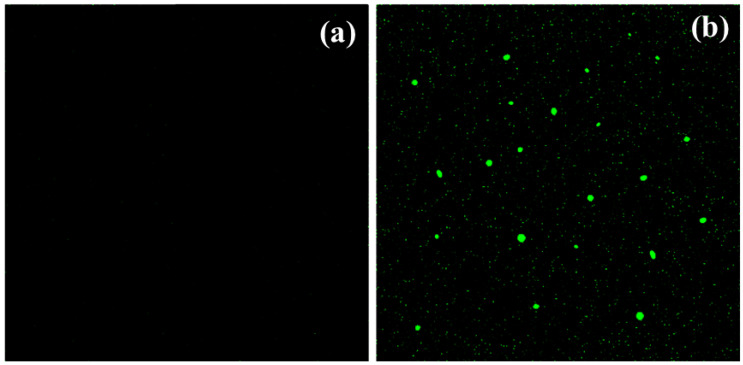
ROS examination (**a**) in the absence and (**b**) presence of [Ni(PLSC-H)_2_H_2_O.

As previously postulated, the enhanced antibacterial and photocatalytic activity of Compounds **1** and **2** might result from reactive oxygen species (ROS) formation. These species include superoxide ions, hydroxyl radicals, and hydrogen peroxide. The experimental and theoretical analyses of the reaction mechanisms involving free radical species are important for understanding their formation and the antioxidant activity of compounds [43,44,45]. ROS can destroy the cell membrane, DNA, and proteins, and ultimately lead to cell death [46]. The formation of ROS in the presence of Compounds **1** and **2** was confirmed by the green fluorescence (excitation wavelength 488 nm) of dichlorofluorescein, which is a product of the reaction between ROS and dichlorofluorescein-diacetate [47]. The *E. coli* bacteria were treated with dichlorofluorescein-diacetate and Compounds **1** and **2**. Figure 8a shows the absence of green fluorescence in the case of untreated *E. coli*. The appearance of green fluorescence is evident in Figure 8b, representing *E. coli* with Compound **1**. These results prove the assumption that ROS can be formed in the presence of Compound **1**. A similar thing was observed for Compound **2**, as shown in Figure S6. Consequently, the outcomes of the present work confirm that ROS were produced within the cell due to the presence of the title compounds and they play a vital role in the inhibition of the hazardous activities of the various microorganisms [37,47].

### 2.9. Cyclic Voltammetry of Compounds ***1*** and ***2***

**Figure 9:**
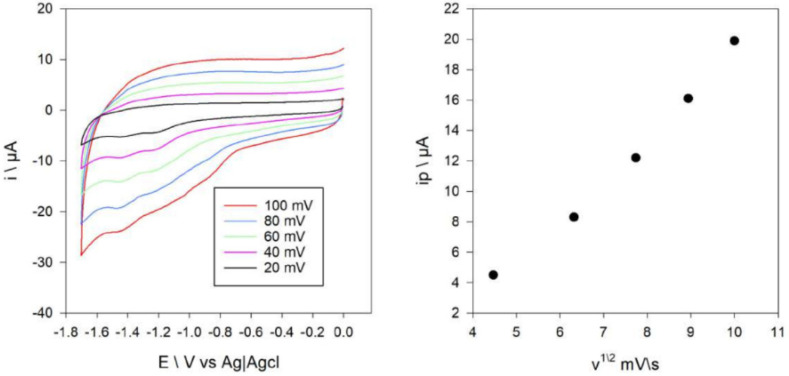
Cyclic voltammograms of 0.35 mM of Compound **1** at a carbon electrode, at different scan rates in DMF containing 0.1 M [NBu4][BF4] (**left**), and plot of ip red for the Ni(II)/Ni(I) and Ni(I)/Ni(0) couple versus the square-root of the scan-rate (**right**).

The electrochemical behavior of the compounds was assessed using the cyclic voltammetry at the vitreous carbon electrode under nitrogen in 0.2 M solution of [Bu_4_N][BF_4_] in DMF. Two waves were observed in the cyclic voltammogram of Compound **1** at potentials of −1.28 and 1.42 V vs. Ag^+^/AgCl (Figure 9). These peaks correspond to the reduction of Ni(II) to Ni(I) and Ni(I) to Ni(0). Figure S7 shows a cyclic voltammogram of Compound **2**, where two peaks are shown at −0.92 and −1.48 V vs. Ag^+^/AgCl. These reduction waves are assigned as Cu(II)/Cu(I) and Cu(I)/Cu(0). The diffusion coefficients of Compounds **1** and **2** were estimated from the Randles–Sevcik equation [48] as the dependence of ip on the scan rate was linear (Figure 9 and Figure S7). The intercept of the mentioned plots was close to zero, which proved that other chemical reactions except for one-electron transfer did not occur. These coefficients were 4 × 10^−3^ cm^2^ s^−1^ and 6.4 × 10^−3^ cm^2^ s^−1^ for Compounds **1** and **2**, respectively. The electrochemical reduction of the ligand was not observed under these experimental conditions.

### 2.10. Magnetic Moment Measurements and Molar Conductivity

The magnetic moments were determined for both complexes. These values were 2.3 and 3.1 BM for Compounds **1** and **2**, respectively, which showed that both compounds were magnetic. The molar conductivities of compounds were 183 (Compound **1**) and 160 (Compound **2**) S cm^2^ mol^−1^. These values were substantially higher than for the 1:1 type of electrolytes, due to the presence of double-charged cations and anions formed in the solution [3].

### 2.11. Molecular Docking

Janus kinase (JAK) can be classified in the nonreceptor tyrosine kinase family, which helps in the transmission of signals initiated by extracellular factors through transmembrane receptors into the cells [49] of mammals. The catalytic domain (JH1) or phosphotyrosine kinase (PTK) domain is essential for tyrosine kinase physiological activity. Selective JAK1 inhibitors can be used for potential therapies for rheumatoid arthritis, other inflammatory diseases, immunologic disorders, and cancers, with minimal side effects such as anemia. An efficient way to inhibit JAK1 is by targeting the ATP binding site inside the JH1 domain [50].

**Figure 10:**
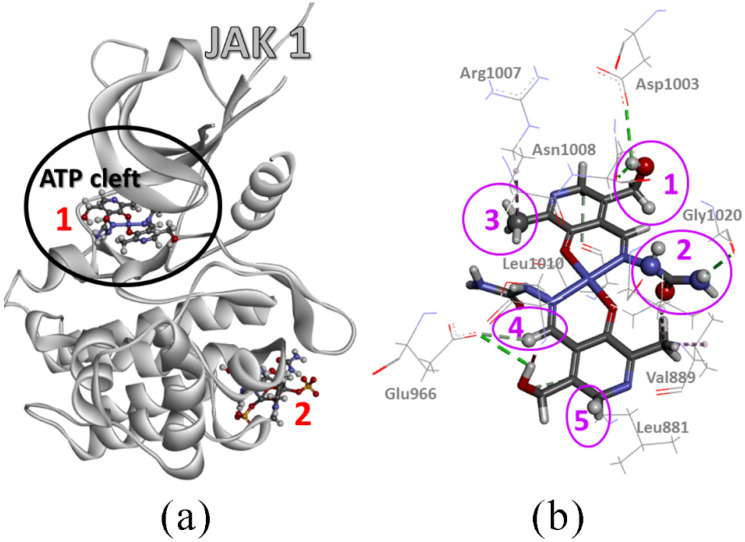
(**a**) Adduct of JAK1 protein (PDB: 6SM8) with Ni(II) complex (Compound **1**) and Cu(II) complex (Compound **2**) (receptor protein is displayed as grey ribbon, while ligand complexes are in a ball and stick representation, colored according to element type) and (**b**) intermolecular interactions (dashed lines) of Ni(II) complex (stick representation) inside ATP cleft of the 6SM8 protein structure (line representation is reserved for amino acid side chains; to stress each functional group of the Ni(II) complex that accomplishes the interactions inside the cleft, the ball-stick representation and pink spheres are used; the numbers are for interacting functional groups of the Ni(II) complex: group 1—CHOH; group 2—NHCONH_2_; group 3—CH_3_; group 4—C_AR_H; and group 5—C_AR_H_2_).

After numerous attempts to position Compound **2** in the active pocket of JAK1, this compound failed to bind inside the ATP cleft (Figure 10), but rather did so at the periphery of the 6SM8 protein structure. Therefore, the formed adduct has a low binding energy (around −17 kJ mol^−1^) and could easily dissociate. Compound **2** only forms a few hydrogen bonds via its –OH, –NH, and sulfate groups. The sulfate group also forms a few unfavorable repulsive interactions.

On the other hand, Compound **1** exclusively has affinity towards the ATP cleft. Its centrosymmetric nature with the Ni(II) as the center of symmetry allows the same interactions for both halves of the molecule. Consequently, four different orientations exist with almost the same binding energies, i.e., close to −40 kJ mol^−1^. Pretty high binding energy values are achieved due to the simultaneously formed hydrogen bonds and close-contact alkyl interactions. Classic hydrogen bonds are formed between –OH, –NH, –CO–, and –C=O groups with Leu881, Ser963, Glu966, Asp1003, Asn1008, Gly1020, and Asp1021 side chains. Carbon hydrogen bonds are formed between -C_AR_H group and Leu881, Glu883, Gly962, Ser963, Glu966, and Gly1020 side chains. Close contact alkyl-alkyl interactions form a -CH_3_ group with Leu881, Val889, Leu1010, and Arg1007 side chains. Compared to the Ni(II) complex, the inhibitor from the 6SM8 protein structure is placed at the same position, achieving weak hydrogen bonds (π-alkyl, halogen-π, π-S, and carbon-hydrogen bonds) and a lower number of strong hydrogen bonds with the same or similar amino acid side chains. It can be concluded that this compound shows promising activity toward the selected protein.

## 3. Materials and Methods

The chemicals used for the preparation of ligands and all other measurements were obtained from Sigma Aldrich and used without purification. The ligands were prepared following the previously described procedure [51].

### 3.1. Synthesis of [Ni(PLSC-H)_2_]H_2_O

A mixture of 0.27 g (1 mmol) of PLITSC and 0.28 g (1 mmol) of NiCl_2_·6H_2_O was dissolved in 15 mL of water. The reaction mixture was then warmed. The warm suspension was separated, cooled for approximately 10 h, after which green crystals formed. Crystals were rinsed off with ethanol. Yield: 0.16 g (65%).

### 3.2. Synthesis of [Cu(PLSC)(SO_4_) (H_2_O)]_2_·2H_2_O

A mixture of 0.21 g (0.1 mmol) PLSC·3H_2_O and 0.5 g (0.5 mmol) CuSO_4_·5H_2_O was dissolved in 15 mL of water and the reaction mixture was warmed until the color of the solution changed to clear brown–green. After prolonged heating (2 h) and a change in pH (diluted HCl added), the change of color was observed. When complete clarity of the solution (which could be prefiltered) was achieved, the suspension was left to crystallize at room temperature. After a few days, crystallization had begun, and clear green monocrystals were visible. The obtained crystals were filtered off and dried in a vacuum. Yield: 0.24 g (88%).

### 3.3. IR Spectroscopy, Magnetic Susceptibility, Molar Conductivity, and Elemental Analysis

The IR spectra were recorded on a Thermo Nicolet (NEXUS 670FT-IR, Waltham, MA, USA) instrument, Department of Chemistry, University Hail, by preparing a KBr pastille (compound: KBr = 1 mg: 150 mg) in the range between 4000 and 750 cm^−1^. The molar susceptibilities of the obtained compounds were measured on a magnetic susceptibility balance (Johnson Matthey Chemicals Limited, London, England) at room temperature. The molar conductivities of 1 × 10^−3^ M solutions were measured on a Jenway 4010 conductivity meter. Elemental (C, H, N) analysis of air-dried samples was carried out using standard micromethods in the Centre for Instrumental Analysis, Faculty of Chemistry, Belgrade.

Anal. Calcd. [Ni(PLSC-H)_2_]·H_2_O (C_18_H_26_N_8_O_8_Ni): C 39.92%, H 4.81%, N 20.69%. Found: C 39.50%, H 5.01%, N 21.06%.

Anal. Calcd. [Cu(PLSC)(SO_4_)(H_2_O)]_2_·2H_2_O (C_18_H_36_N_8_O_19_S_2_Cu_2_): C 25.12%, H 4.19%, N 13.00%. Found: C 25.45%, H 4.30%, N 13.09%.

### 3.4. X-ray Analysis

Single crystals with the following dimensions (0.12 × 0.09 × 0.17) (Ni(II) complex) and (0.15 × 0.08 × 0.15) (Cu(II) complex) were examined. The X-ray measurements were performed on a single crystal of the complex was mounted on glass fiber and examined at 296 K. Bruker D8 Venture APEX diffractometer equipped with Photon 100 area detector using graphite-monochromate Mo-Kα radiation [λ = 0.71073 Å] was used for these measurements. Based on the difference map, the location of hydrogen atoms was determined. Hydrogen atoms bound to carbon were initially positioned geometrically, while the hydrogen atoms for the coordinated water molecules were found through the difference map. All hydrogen positions and isotropic displacement parameters were then refined in a separate cycle. Hydrogen positions were checked for feasibility by examination of the hydrogen-bonding network. Crystallographic data for the complexes were deposited in the Cambridge Crystallographic Data Centre (CCDC, 12 Union Road, Cambridge, UK; e-mail: depos-it@ccdc.cam.ac.uk), CDC deposition number is 2163557 and 2152708. Crystal data collection and structure refinement are given in Table 1.

### 3.5. Hirshfeld Surface Analysis

Hirshfeld surface analysis is a method for the quantitative investigation of the interaction governing the crystal structure. This type of analysis was performed in the Crystal-Explorer program package [52]. Two distances are shown on the Hirshfeld surface: one representing the distance between two nearest nuclei (d_e_) and the second being the distance from nuclei to the external surface (d_i_) [53,54,55]. The normalized distance (d_norm_) is then represented by various colors, namely red, white, and blue if the distance is shorter, equal, or longer than the van der Waals radii separation between atoms. The normalized distance in this contribution is shown in the range between −0.4939 au (red) and 1.1572 au (blue). The fingerprint plots for the various combinations of atoms are given in the Supplementary information.

### 3.6. Theoretical Calculations

The structures of both complexes were analyzed in the Gaussian Program package (Gaussian 09, Revision C.01) [56] starting from the crystallographic structure. The global hybrid Generalized Gradient Approximation (GGA) functional B3LYP [57] was used in the conjunction with 6-31+G(d,p) basis set [58] for H, C, N, and O atoms and LanL2DZ basis set [59,60] for Cu and Ni. This complex was selected due to its unique structural features. The optimization was performed without any geometrical constraints and the absence of imaginary frequencies showed that the minima on the potential energy surface were found. The natural bond orbital analysis [61,62,63] was used to investigate the stabilization interactions within structures of the ligand and complex that are responsible for the stability of the structure.

### 3.7. Photocatalytic Evaluation

The photocatalytic activity of Compounds **1** and **2** was determined by following the degradation of a methylene blue dye (MB) in water solution under the presence of UV light. At the start of the experiment, 20 mL of MB solution (10 mg L^−1^) containing 8 mg of complexes was stirred for 30 min in the absence of light to achieve the adsorption–desorption equilibrium. In 15 min intervals, 0.3 mL of solution were taken out and MB concentrations were evaluated by UV-VIS spectroscopy (Thermo Scientific Evolution 220 UV-VIS spectrophotometer, Waltham, MA, USA) at 664 nm after the removal of complexes by centrifuge at 1000× *g* rpms. The concentrations of MB were corrected for the volume change.

### 3.8. Antibacterial Activity of Obtained Complexes

The agar well diffusion method was used to determine the antibacterial properties of the title compounds [64]. To produce bacterial strains, the nutritive broth was incubated at 37 °C for 24 h to achieve a turbidity of 0.5 (McFarland standard) [65,66]. The bacteria selected for this study include Gram-negative and Gram-positive bacteria such as *Escherichia coli* (*E. coli*) and *Bacillus subtilis* (*B. subtilis*). For this purpose, uniform 8 mm equidistant holes in the Petri plate were made by using a sterile cork borer. The solution of obtained complexes with a concentration of 2 mg mL^−1^ was prepared and sonicated for 30 min. After that, 50 μL of various concentrations from 0.25 to 2 mg mL^−1^ were injected into each hole to assess the zone of inhibition at 35 °C. A 50 μL (2 mg mL^−1^) dilution of kanamycin was utilized as a control sample. Finally, after a 24 h incubation period at 37 °C, the diameter of the inhibitory zone was measured. The experiment was repeated three times, with the findings presented as mean and standard deviation.

### 3.9. Minimum Inhibitory Concentration (MIC)

The serial dilution procedure revealed the MIC behavior of complexes against the selected microorganisms. Solutions of various concentrations (20 to 120 μg mL^−1^) were prepared. After that, 1 mL of each solution was taken and mixed with 1 mL of bacterial suspension (*E. coli* and *B. subtilis*) and 0.5 McFarland turbidity standards in an uncontaminated test tube. Finally, the test tubes were placed in an incubator and kept at 37 °C for 24 h. The control group, on the other hand, included growth media and bacteria. The determination of MIC for both complexes was performed three times.

### 3.10. Determination of ROS

The most important agent against bacteria growth is the intracellular generation of reactive oxygen species (ROS) in the presence of the obtained compounds. The DCFH-DA (2, 7-dichloro-dihydro-fluoresceindi-acetate) assay was utilized to show that both compounds produce ROS intracellularly. Obtained complexes and the tested bacterial strain (*B. subtilis*), were incubated for 3 h at a speed of 300 rpm. After that, the bacterial cell suspension was collected by centrifuging for 5 min at 10,000× *g* rpm. Phosphate buffer saline (PBS) was also used to wash the pellet that was obtained. The pellet was then suspended in 1 mL PBS and treated for 40 min with 1 mL of 20 mM DCFH-DA reagent. The cells were rinsed three times with the buffer solution PBS to remove the excess dye from the outer surface. Finally, a fluorescent microscope (Olympus 1 × 51) was utilized to obtain a fluorescence image of the aforementioned solution at wavelengths of 488 nm and 535 nm, respectively [24,67,68].

### 3.11. Cyclic Voltammetry

Cyclic voltammetry experiments were performed on an Au-tolab PGSTAT 128 potentiostat. The electrochemical cell contained 5 mL of 0.2 M solution of obtained compounds in DMF. Before the measurements, the solutions were degassed with nitrogen. A conventional three-electrode arrangement was employed, consisting of a vitreous carbon working electrode (CPE) (0.07 cm^2^), a platinum wire as the auxiliary electrode, and Ag/AgCl as a reference electrode.

### 3.12. Molecular Docking

The target protein for the study was obtained on the Swiss Target Prediction website [69], based on the similarity between the native ligand and the available proteins. The optimized geometries of the investigated nickel(II) and copper(II) complexes at the mentioned level of theory were used as ligands, while the PDB file of the JAK1 crystal structure (code 6SM8 [49]) was used as a target. When a computational docking is initiated, the AutoDockVina engine [70] automatically runs the algorithm Iterated Local Search global optimizer, Broyden-Fletcher-Goldfarb-Shanno (BFGS) method for the local optimization, and Vina hybrid scoring function (empirical + knowledge-based function) inspired by the X-Score [71]. To find the most preferable binding site in the target protein for each ligand, the docking calculation was performed on the whole protein. The greed box size was 120 × 100 × 100, with the center at the point (−8.947, 54.274, 9.679) and the step size 1 Å. The necessary preparations for molecular docking were performed in AutoDockTools4 [71] software, while BIOVIA Discovery Studio was used for the visualization and data analysis of the docking results [72].

## 4. Conclusions

The pyridoxal-semicarbazone ligand was in the monoanionic form in [Ni(PLSC-H)_2_] H_2_O (Compound **1**) and neutral form in [Cu(PLSC)(SO_4_)(H_2_O)]_2_·2H_2_O (Compound **2**), as shown by X-ray crystallography and IR spectroscopy. The highest contribution to interactions between monomeric units of the compound is from the H∙∙∙H (44.8%) and H∙∙∙O (23.3%) contacts for Compound **1** and H∙∙∙O (56.5%) contacts for Compound **2**, which is a consequence of the large number of oxygen atoms in the ligand structure. The crystallographic structures, optimized at the B3LYP/6-31+G(d,p) level of theory for H, C, N, O, and S atoms and B3LYP/LanL2DZ for Ni/Cu, have mean absolute error values for the bond lengths and angles of the ligand equal to 0.014 Å and 2.42°, respectively, which proved the applicability of the chosen level of theory. The interactions with carbonyl *O* were the strongest among all donor atoms, as shown in the NBO analysis. The reduction of the methylene blue concentration was 90 and 98%, with reaction rates of 2.2 × 10^−4^ and 3.6 × 10^−4^ s^−1^ in the presence of Compounds **1** and **2**, respectively. The inhibition zone towards *E. coli* and *S. aureus* increased with the increase in concentrations of both compounds, although Compound **2** had a higher antibacterial activity. The MIC values of the obtained complexes were comparable to the positive control, chloramphenicol. The antibacterial activity of these compounds is probably due to the formation of reactive oxygen species, as shown in the experiment with dichlorofluorescein-diacetate, which led to the formation of fluorescent dichlorofluorescein. The cyclic voltammograms of Compound **1** had peaks at −1.28 and 1.42 V vs. Ag^+^/AgCl, which correspond to the reduction of Ni(II) to Ni(I) and Ni(I) to Ni(0). On the other hand, the cyclic voltammograms of Compound **2** had peaks at −0.92 and −1.48 V vs. Ag^+^/AgCl, which were assigned as Cu(II)/Cu(I) and Cu(I)/Cu(0). According to molecular docking calculations, the Ni(II) complex easily binds to the ATP-binding site of the JAK1 protein with a binding energy of −40.6 kJ mol^−1^. The Cu(II) binuclear complex cannot be recognized as an inhibitor, as it binds outside of the active pocket with a binding energy of −17.2 kJ mol^−1^.

## Figures and Tables

**Table 1:** Crystal data and structure refinement details of Compounds **1** and **2**.

Empirical Formula	C_18_H_26_N_8_NiO_8_	C_18_H_36_N_8_O_19_S_2_Cu_2_
Formula weight	541.16	859.77
Temperature	123 K	123 K
Wavelength	1.54184	0.71073
Crystal System	I 2/a	P-1
Space group	monoclinic	triclinic
Volume	2215.48 (6)	1492.58 (3)
Unit cell dimension	a = 12.4040 (2)	a = 9.2533 (1)
	b = 13.5306 (2)	b = 9.5788 (1)
	c = 13.5689 (2)	c = 17.0995 (2)
	α = 90	α = 83.858 (1)
	β = 103.383 (1)	β = 82.899 (1)
	γ = 90	γ = 85.791 (1)
Z	4	2

**Table 2:** Zone of inhibition of Compounds 1 and 2 and standard drug against tested bacteria.

Microorganisms	Concentration (mg mL^−1^)	Zone of Inhibition (mm)			
		Compound 1	Compound 2	Positive Control	Negative Control
*E. coli*	0.25	10 ± 0.1	9 ± 0.2	15 ± 0.3	Zero inhibition
	0.5	14 ± 0.2	13 ± 0.2		
	1	17 ± 0.3	18 ± 0.3		
	2	21 ± 0.3	24 ± 0.4		
*S. aureus*	0.25	12 ± 0.2	11 ± 0.1	16 ± 0.2	Zero inhibition
	0.5	16 ± 0.2	15 ± 0.2		
	1	21 ± 0.3	20 ± 0.3		
	2	24 ± 0.4	26 ± 0.3		

**Table 3:** Zone of inhibition of Compounds **1** and **2** and standard drug against tested bacteria.

Compound	Bacterium	Concentration (μg mL^−1^)				
		60	50	40	30	20
Compound **1**	*S. aureus*	−	−	−	−	+
	*E. coli*	−	−	−	+	+
Compound **2**	*S. aureus*	−	−	−	−	−
	*E. coli*	−	−	−	−	+
Chloramphenicol	*S. aureus*	−	−	−	−	−
	*E. coli*	−	−	−	−	−

− represents strong inhibition of bacteria; + represents partial inhibition of bacteria.

## Data Availability

Not applicable.

## Supplementary Materials

The following supporting information can be downloaded at: https://www.mdpi.com/article/10.3390/molecules27196322/s1, Table S1: Crystal data and structure refinement details of Compounds **1** and 2; Figure S1: IR spectra of PLSC; Figure S2: The fingerprint plots for the most important contacts by elements for [Ni(PLSC-H)_2_] H_2_O; Figure S3: The fingerprint plots for the most important contacts by elements for [Ni(PLSC-H)_2_] H_2_O; Table S2: The experimental and theoretical bond lengths of ligand (numbering scheme shown below); Table S3: The experimental and theoretical bond angles of ligand (numbering scheme shown below); Figure S4: UV-VIS spectrum of ligand; Figure S5: Kinetic curves for the reduction of MB by Compounds **1** and **2**; Figure S6: Determination of ROS (a) represents the untreated *E. coli* and (b) represents the *E. coli* treated with [Cu(PLSC)(SO_4_)(H_2_O)]_2_·2H_2_O; Figure S7: Cyclic voltammograms of 0.25 mM Compound **2** at carbon electrode, at different scan-rates in DMF containing 0.1 M [NBu_4_][BF_4_] (left) and plot of ip red for the Ni(II)/Ni(I) and Ni(I)/Ni(0) couple versus the square-root of the scan-rate (right).
