# Supplementary material for: Synthesis, Crystal Structure, Theoretical Calculations, Antibacterial Activity, Electrochemical Behavior, and Molecular Docking of Ni(II) and Cu(II) Complexes with Pyridoxal-Semicarbazone
Source: Molecules. 2022 Sep 26;27(19):6322. doi: 10.3390/molecules27196322 (PMC9570950; doi:10.3390/molecules27196322)
Supplement: Supplementary file 1 [file molecules-27-06322-s001.zip › molecules-1926221-supplementary.pdf]

Supplementary Information for:

Synthesis, Crystal Structure, Theoretical Calculations, Antibacterial Activity, Electrochemical Behavior, and Molecular Docking of Ni(II) and Cu(II) Complexes with Pyridoxal-Semicarbazone

Violeta Jevtovic [1], Njood Alsammari [1], Salman Latif [1], Abdulmohsen Khalaf Dhahi Alsukaibi [1], Jamal Humaidi [1], Tahani Y. A. Alanazi [1], Fahad Abdulaziz [1], Samah I. Matalka [1], Nebojša Đ. Pantelić [2], Milica Marković [3], Aleksandra Rakić [3] and Dušan Dimić [3,*]

[1] Department of Chemistry, College of Science, University of Hail, Ha'il 81451, Saudi Arabia
[2] Department of Chemistry and Biochemistry, Faculty of Agriculture, University of Belgrade, Nemanjina 6, 11080 Belgrade, Serbia
[3] Faculty of Physical Chemistry, University of Belgrade, Studentski trg 12-16, 11000 Belgrade, Serbia

Table S1. Crystal data and structure refinement details of compounds **1** and **2**.

**Bond length [A]**		**Angle [°]**	
Compound **1**	Compound **2**	Compound **1**	Compound **2**
Ni1–O12 2.101 (2)	Cu–O7 1.901 (1)	O12–Ni1–O16 167.31 (6)	O7–Cu1–O6 165.94 (6)
Ni1–O16 1.981 (1)	Cu1–O6 1.959 (1)	O12 –Ni1–N9 78.64 (5)<br>O16–Ni1–N9′ 89.16 (6)	O7–Cu1–O1 94.42 (6)
Ni1–N9 2.003 (2)	Cu1–O1 1.936 (1)	O12–Ni1–O12′ 87.68 (6)	O7 – Cu1 –O2 86.88 (5)
Ni1 – O12′ 2.101 (2)	Cu1–O2 2.262 (2)	O12′–Ni1–O16′ 90.38 (5)	O7 – Cu1 – N1 91.22 (6)
Ni1 – O16′ 1.981 (2)	Cu1–N1 1.967 (1)	O12′–Ni1–N9′ 96.89 (6)	O6 –Cu1 –O1 91.46(6)
Ni1-N9′ 2.003 (2)	Cu1–O14 2.94 (2)	O12–Ni1–O16′ 90.38 (5)	

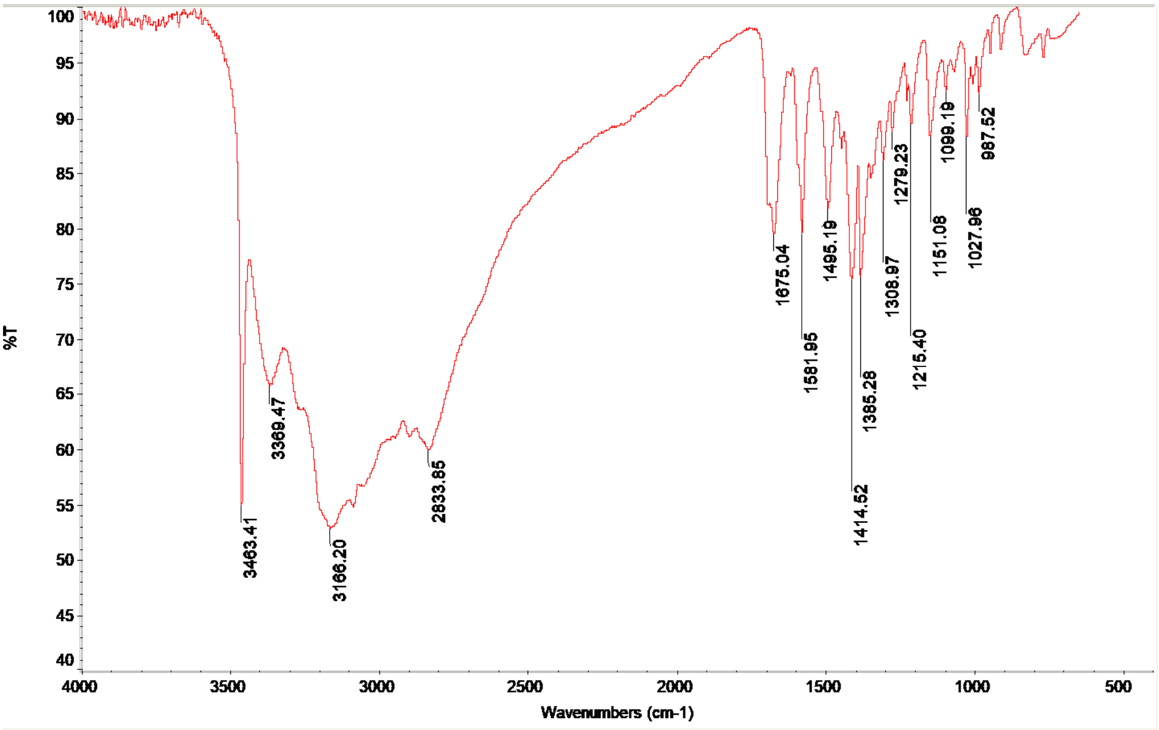

Figure S1. IR spectrum of PLSC

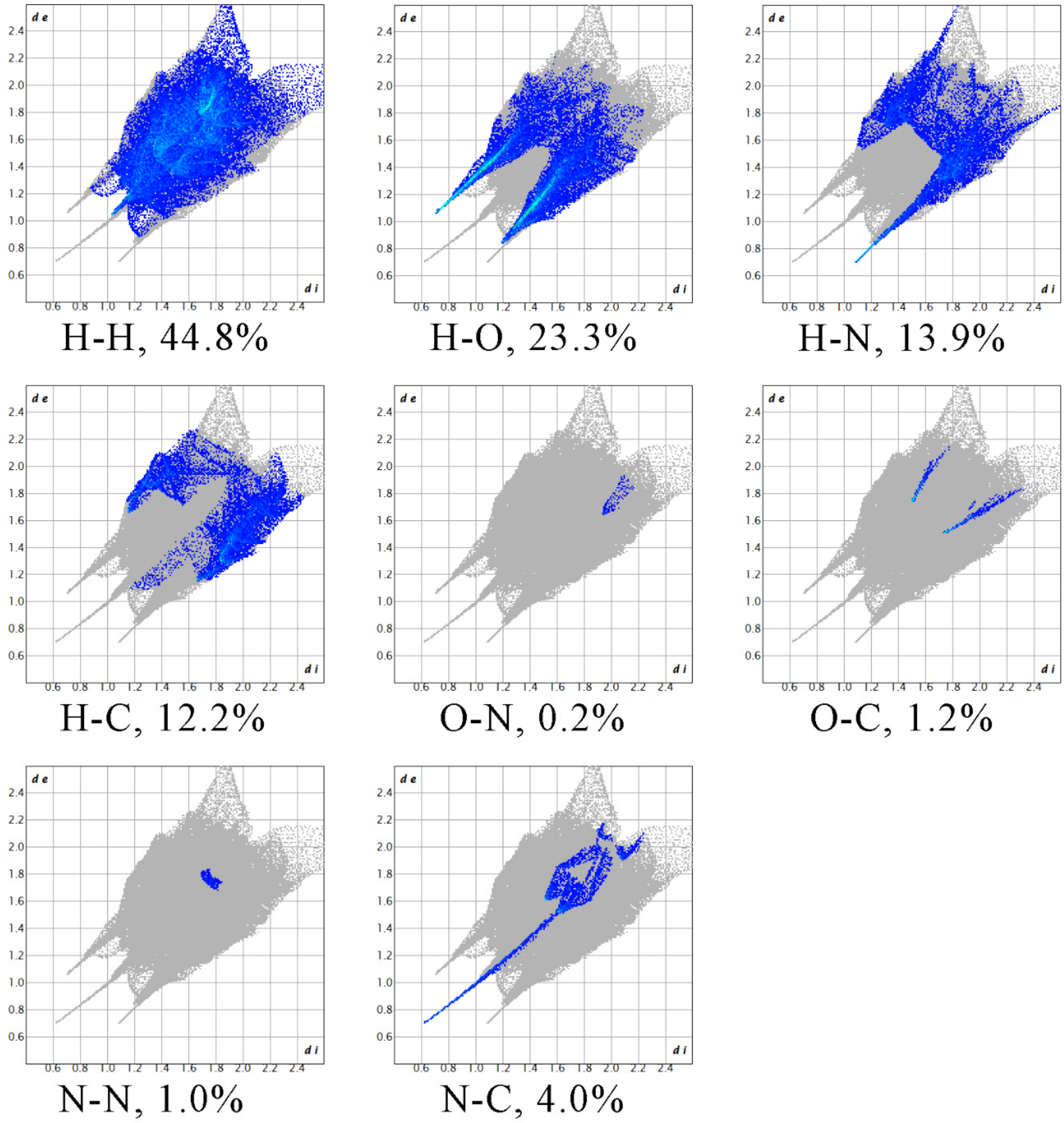

Figure S2. The fingerprint plots for the most important contacts by elements for [Ni(PLSC-H)$_2$]$\cdot H_2O$

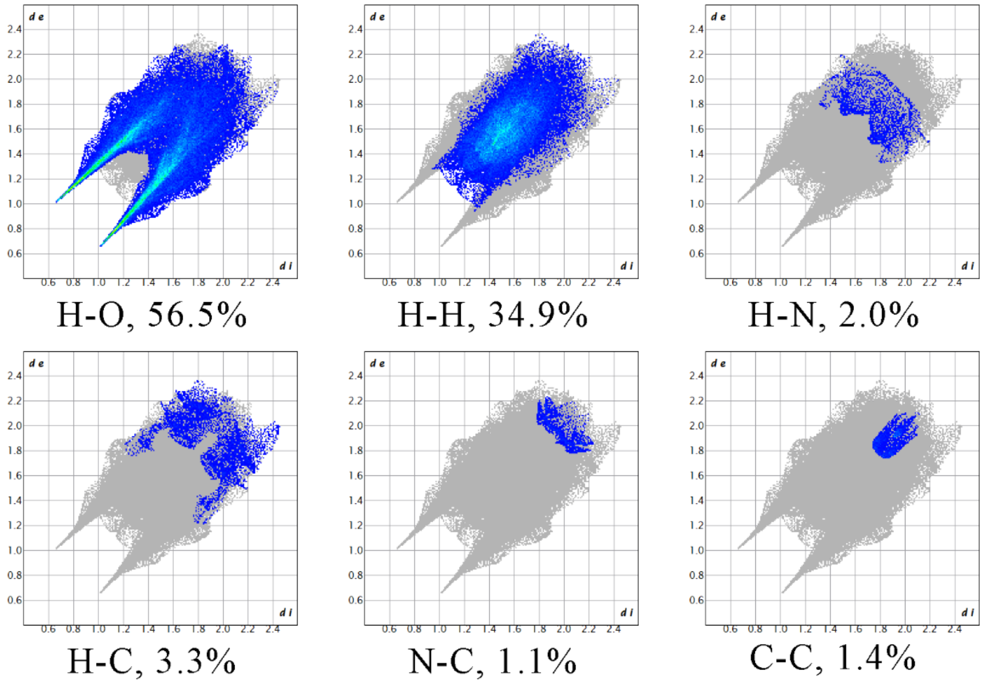

Figure S3. The fingerprint plots for the most important contacts by elements for [Ni(PLSC-H)$_2$]·H_2O

Table S2. The experimental and theoretical bond lengths of ligand (numbering scheme shown below)

Bond	Experimental [Å]	Theoretical [Å]
C41-C42	1.490	1.488
C42-N33	1.331	1.342
N33-C46	1.356	1.362
C46-C45	1.372	1.373
C45-C44	1.423	1.430
C44-C43	1.430	1.436
C43-O7	1.297	1.295
C45-C47	1.520	1.520
C44-C50	1.456	1.452
C50-N34	1.290	1.305
N34-N35	1.368	1.383
N35-C51	1.365	1.407
C51-O6	1.264	1.232
C51-N36	1.318	1.364
C47-O5	1.432	1.415
MAE [Å]		0.014

Table S3. The experimental and theoretical bond angles of ligand (numbering scheme shown below)

Angle	Experimental [º]	Theoretical [º]
C41-C42-N33	119.98	118.44
C41-C42-C45	120.53	123.28
C42-N33-C46	123.91	124.63
N33-C46-C45	120.31	120.03
C46-C45-C44	119.22	119.38
C46-C45-C47	117.37	117.38
C45-C47-O5	113.77	113.41
C45-C44-C43	119.23	118.89
C44-C43-C42	117.71	118.63
C45-C44-C50	119.01	115.62
C43-C44-C50	121.69	124.81
C44-C50-N34	121.43	127.77
C50-N34-N35	119.31	111.55
N34-N35-C51	114.61	117.24
N35-C51-O6	120.16	122.94
C35-C51-N36	117.33	111.84
O6-C51-N36	122.50	125.17
MAE [º]		2.42

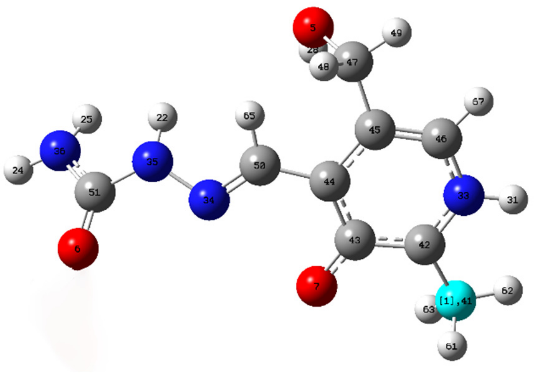

Numbering scheme for the comparison of experimental and theoretical bond lengths and angles.

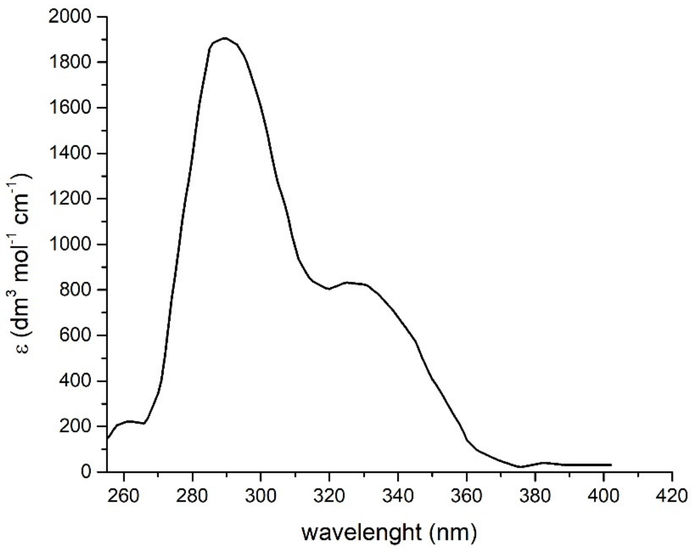

Figure S4. UV-Vis spectrum of ligand

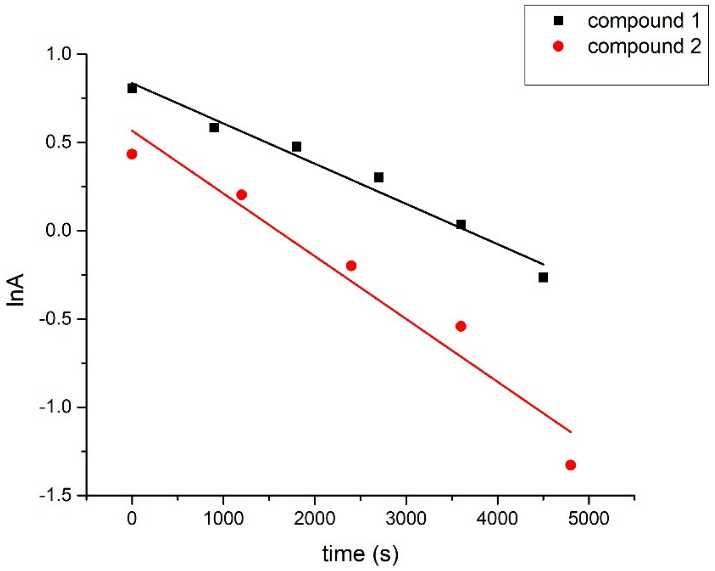

Figure S5. Kinetic curves for the reduction of MB by compounds **1** and **2**

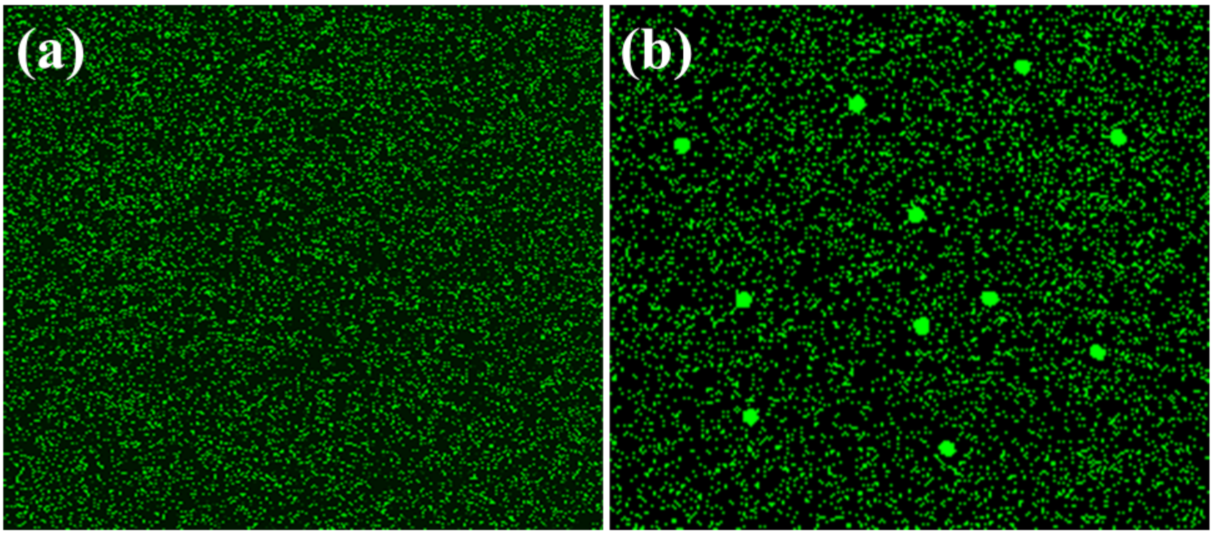

Figure S6. Determination of ROS (a) respresents the untreated E. coli and (b) respresents the E. coli treated with [Cu(PLSC)(SO4)(H2O)]2·2H2O.

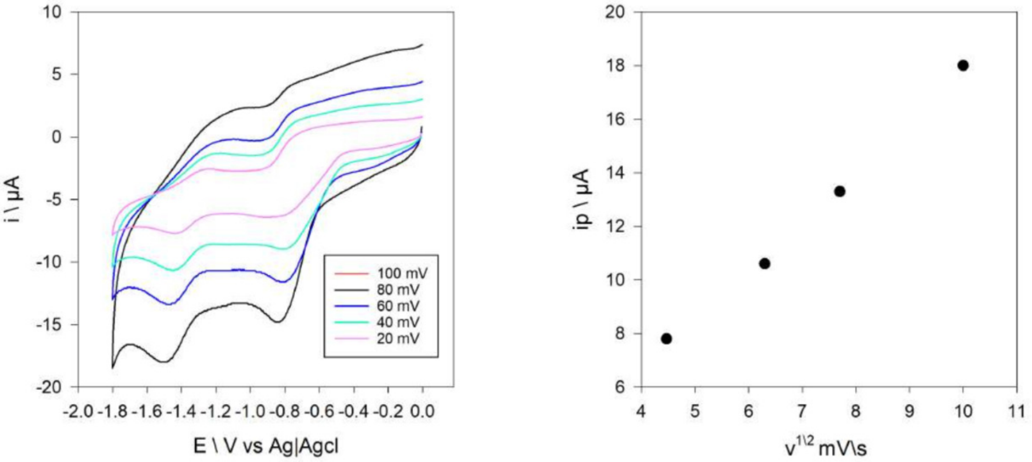

Figure S7. Cyclic voltammograms of 0.25 mM compound **2** at carbon electrode, at different scan-rates in DMF containing 0.1M [NBu4][BF4] (left) and plot of ip red for the Ni(II)/Ni(I) and Ni(I)/Ni(0) couple versus the square-root of the scan-rate (right).
